# Fundus Image-Based Eye Disease Detection Using EfficientNetB3 Architecture

**DOI:** 10.3390/jimaging11080279

**Published:** 2025-08-19

**Authors:** Rahaf Alsohemi, Samia Dardouri

**Affiliations:** 1 Department of Computer Science, College of Computing and Information Technology, Shaqra University, Shaqra 11911, Saudi Arabia; 2 InnoV’COM Laboratory-Sup’Com, University of Carthage, Ariana 2083, Tunisia

**Keywords:** EfficientNetB0, eye disease classification, diabetic retinopathy, cataract, glaucoma, deep learning, fundus images, image augmentation, CNN, medical image analysis

## Abstract

Accurate and early classification of retinal diseases such as diabetic retinopathy, cataract, and glaucoma is essential for preventing vision loss and improving clinical outcomes. Manual diagnosis from fundus images is often time-consuming and error-prone, motivating the development of automated solutions. This study proposes a deep learning-based classification model using a pretrained EfficientNetB3 architecture, fine-tuned on a publicly available Kaggle retinal image dataset. The model categorizes images into four classes: cataract, diabetic retinopathy, glaucoma, and healthy. Key enhancements include transfer learning, data augmentation, and optimization via the Adam optimizer with a cosine annealing scheduler. The proposed model achieved a classification accuracy of 95.12%, with a precision of 95.21%, recall of 94.88%, F1-score of 95.00%, Dice Score of 94.91%, Jaccard Index of 91.2%, and an MCC of 0.925. These results demonstrate the model’s robustness and potential to support automated retinal disease diagnosis in clinical settings.

## 1. Introduction

A vital component of everyday existence and quality of life, the human eye is a highly complex sensory organ that is vital to the capture and interpretation of visual information. Millions of people around the world, however, suffer from eye-related conditions that can cause irreversible visual damage or total blindness if they are not identified or treated. The World Health Organization (WHO) estimates that approximately 2.2 billion people worldwide suffer from blindness or vision impairment, with at least 1 billion of these instances being avoidable or untreated. Cataracts, diabetic retinopathy, glaucoma, and other degenerative illnesses are among the most prevalent eye ailments. These conditions continue to be a significant public health concern, especially in underprivileged areas and among aging populations [1]. Effective intervention depends on early and accurate detection of eye illnesses, but traditional diagnostic methods mostly rely on skilled ophthalmologists manually assessing retinal pictures, which takes a lot of time and resources. Delays in diagnosis can result in permanent damage and accelerated illness progression in areas with limited access to specialized care. By using medical image analysis and artificial intelligence (AI) techniques, computer-aided diagnosis (CAD) systems have become a promising solution to these problems. These systems help ophthalmologists identify and categorize eye conditions from fundus photos or optical coherence tomography (OCT) scans [2,3].

Medical imaging has undergone a tremendous revolution thanks to recent developments in deep learning, especially in the areas of disease identification and categorization. In a variety of applications involving object detection, picture recognition, and medical diagnostics, Convolutional Neural Networks (CNNs) have shown impressive performance. With fewer parameters and lower computational costs, EfficientNet, a family of highly efficient and scalable CNN architectures, has demonstrated state-of-the-art performance in a number of classification benchmarks [4]. Specifically, EfficientNetB3 provides a good compromise between accuracy and model complexity, which makes it ideal for implementation in actual healthcare systems.

Deep learning models face a number of obstacles in medical imaging applications, notwithstanding their potential. Retinal pictures frequently differ in terms of noise, illumination, resolution, and quality based on the patient population and the equipment being utilized. Additionally, the effectiveness of traditional classifiers may be hampered by the intra-class diversity within illness categories and the inter-class similarities between specific eye disorders. Therefore, enhancing generalizability and diagnostic accuracy requires strong data preparation, augmentation, and model fine-tuning. Using high-resolution retinal pictures, this study suggests a deep learning framework based on EfficientNetB3 for multi-class categorization of eye illnesses. The suggested model attempts to produce accurate diagnostic predictions by utilizing transfer learning, data augmentation, and an efficient training pipeline that makes use of cosine annealing learning rate scheduling and categorical cross-entropy loss. A publicly accessible Kaggle dataset with tagged photos of healthy eyes, diabetic retinopathy, cataracts, and glaucoma was used to train and validate the model. By providing precise, quick, and scalable diagnostic tools that can be included in telemedicine platforms and remote healthcare services, our main goal is to improve clinical decision support systems. This study shows how well EfficientNetB3 distinguishes between various eye disease categories through a thorough evaluation using performance metrics like accuracy, precision, recall, F1-score, and the Matthews Correlation Coefficient (MCC). The suggested method has the potential to support early screening, disease monitoring, and resource-efficient healthcare delivery by bridging the gap between AI research and clinical practice, especially in low- and middle-income nations with a shortage of ophthalmology specialists.

## 2. Related Works

In modern ophthalmology, the ability to accurately classify eye illnesses using medical imaging is essential for reducing vision loss and facilitating early intervention. A common non-invasive imaging technique for seeing the retina for diagnostic reasons is fundus photography. Research into creating automated systems that use deep learning and machine learning to reliably and accurately classify retinal pictures has accelerated in recent years. Historically, the field was dominated by traditional computer vision approaches that relied on statistical classifiers and handmade features. However, when used on big, diverse datasets, these approaches frequently lacked scalability and reliability [1].

Medical image categorization has greatly benefited from deep learning, especially convolutional neural networks (CNNs). For the classification of diabetic retinopathy (DR), glaucoma, cataracts, and other retinal disorders, a number of designs have been put out and assessed. On retinal fundus photos, for example, Ting et al. [2] created a deep learning algorithm for DR identification that performed at an ophthalmologist level. Similarly, for multistage DR classification, Rajalakshmi et al. [3] used a CNN model with data augmentation, producing encouraging accuracy and sensitivity scores. Building upon these frameworks, numerous studies have investigated the use of transfer learning to improve classification performance in pre-trained models. Using VGG16 and InceptionV3 models for glaucoma identification, Pratt et al. [4] made a noteworthy contribution and reported better results after fine-tuning on retinal imaging datasets. The authors of [5] achieved greater generalization on unseen test data by combining ensemble learning and DenseNet architectures to classify various eye disorders from fundus images. Because of its compound scaling approach, which strikes a balance between network depth, width, and resolution, the EfficientNet family has recently become a strong alternative. EfficientNetB0–B7 was introduced by Khan et al. [6], who demonstrated that smaller variants, like EfficientNetB3, may perform on par with or better than deeper networks while using less computing power. EfficientNetB3 outperformed conventional CNNs and showed notable gains in accuracy, sensitivity, and F1-score metrics when used for multi-disease retinal categorization in [7].

In order to improve robustness and solve issues like class imbalance and low contrast in retinal pictures, hybrid models have also been developed. The authors of [8] improved performance in differentiating between related illness categories by combining EfficientNet with attention mechanisms to concentrate on important retinal regions. To further improve dependability in clinical contexts, others have used ensemble techniques that combine predictions from several deep learning models [9]. To combat overfitting and enhance convergence, new learning techniques and loss functions have been used concurrently. Techniques like label smoothing and focal loss have proven very useful in managing unbalanced datasets, which are a frequent problem in medical imaging activities [10]. Additionally, learning rate scheduling techniques like cosine annealing have aided in stabilizing training and speeding up convergence, particularly in large-scale models like EfficientNet. Fair comparison across various approaches has been made easier by recent benchmarks on publicly accessible datasets including EyePACS, APTOS, and Kaggle’s Eye Diseases Classification dataset. An EfficientNetB4 model trained on the APTOS dataset, for instance, was used in a study in [11], which showed the network’s resilience to image noise and unpredictability while attaining over 96% classification accuracy. To boost classification performance and interpretability, researchers in [12] combined domain-specific preprocessing procedures, such as blood vessel segmentation and contrast enhancement. The increasing significance of scalable, high-performance deep learning models in ophthalmology is shown by a comparison of different approaches. Even while conventional CNNs are still useful, EfficientNet and other similar architectures show state-of-the-art performance when paired with the right preprocessing, augmentation, and fine-tuning. These developments represent a major step toward the clinical application of automated diagnostic tools, especially in settings with limited resources and restricted access to specialized care. Recent advancements in deep learning have led to the widespread use of EfficientNet-based models, particularly EfficientNetB3, for automated detection of ocular diseases using fundus imagery. Sharma and Kumar [13] proposed a hybrid EfficientNetB3 and SE block model for diabetic retinopathy detection, achieving improved feature representation. Similarly, Patel and Singh [14] introduced a modified EfficientNetB3 architecture for multi-class classification of fundus images, enhancing diagnostic accuracy. Although the core architecture of EfficientNetB3 is a well-established model, our study goes beyond merely applying transfer learning by introducing several critical improvements tailored to the problem of eye disease classification. First, we implement a robust preprocessing pipeline that enhances image quality and consistency, which is essential for reliable feature extraction in medical imaging. Second, we employ an extensive and carefully designed data augmentation strategy that addresses class imbalance and variability in the dataset, leading to better generalization. Third, we incorporate advanced training techniques such as cosine annealing learning rate scheduling and regularization methods to optimize convergence and prevent overfitting. Moreover, our evaluation includes multiple datasets with diverse patient populations and imaging conditions, demonstrating the model’s adaptability and practical utility. These methodological refinements collectively contribute to a more accurate, interpretable, and clinically relevant model compared to previous works that use standard transfer learning pipelines without such customization. Rahman and Alam [15] employed a CNN integrated with EfficientNetB3 for generalized ocular disease classification, while Deshmukh and Joshi [16] demonstrated the effectiveness of transfer learning using a pre-trained EfficientNetB3 for binary DR detection. Obasi and Nwachukwu [17] showcased a robust EfficientNet-based framework for classifying retinal diseases, highlighting its high accuracy and clinical applicability. Iqbal and Rajesh [18] focused on multistage diabetic retinopathy classification using EfficientNetB3, incorporating stage-specific learning strategies. Furthermore, Kumar and Rani [19] explored transfer learning to adapt pretrained models for fundus image analysis, reducing training time while maintaining performance. Divya and Thompson [20] compared multiple CNNs and confirmed the superiority of EfficientNetB3 in early detection tasks, achieving an AUC of 96.73% on the ODIR dataset. Collectively, these studies underscore the growing reliability of EfficientNetB3 in ophthalmic diagnostics. Tashkandi [21] proposed a deep learning-based system for eye disease prediction using retinal images, achieving notable accuracy. Khalid et al. [22] developed CAD-EYE, an advanced multi-disease classification framework that combines feature fusion and fluorescence imaging to enhance interpretability. Wahab Sait [23] introduced an AI-driven classification model tailored for efficient diagnosis of ocular conditions. Acevedo et al. [24] emphasized disease identification through deep learning, demonstrating strong performance on diverse fundus image datasets. Shamsan et al. [25] explored hybrid feature-based classification techniques, showcasing promising results in disease prediction using color fundus images. Additionally, Du et al. [26] presented RETFound, a powerful model for detecting pathological features in retinal OCT scans, reinforcing the potential of deep learning in improving diagnostic precision and clinical workflows across ophthalmology. Recent research efforts have explored various advanced techniques for automated detection of retinal diseases. For example, Wang et al. [27] investigated the use of hyperspectral imaging for the optical identification of diabetic retinopathy, demonstrating the potential of richer spectral data to enhance diagnostic accuracy. While hyperspectral imaging offers promising results, its adoption in routine clinical practice is currently limited by cost and equipment availability. In contrast, our study focuses on leveraging widely accessible retinal fundus images and state-of-the-art deep learning models, aiming to deliver accurate and scalable disease classification suitable for broader clinical deployment. By referencing and building upon recent developments, our work addresses the practical need for efficient and deployable deep learning solutions in ophthalmology. Table 1 presents a comparative analysis of recent methods employed for eye disease classification using various publicly available and private fundus image datasets.

Our current approach utilizes transfer learning from models pretrained on ImageNet, a large-scale natural image dataset. While ImageNet pretraining provides a strong foundation for feature extraction, it does not capture domain-specific patterns inherent in ophthalmic images. Domain-specific pretraining using large ophthalmic datasets, such as those containing retinal fundus images or OCT scans, may further improve the model’s ability to recognize subtle disease-related features, thereby enhancing classification accuracy and generalizability. However, the limited availability of such comprehensive ophthalmic datasets currently constrains this approach. Future research could focus on leveraging domain-adaptive pretraining or self-supervised learning on ophthalmic data to address this limitation.

Although the core architecture of EfficientNetB3 is a well-established model, our study goes beyond merely applying transfer learning by introducing several critical improvements tailored to the problem of eye disease classification. First, we implement a robust preprocessing pipeline that enhances image quality and consistency, which is essential for reliable feature extraction in medical imaging. Second, we employ an extensive and carefully designed data augmentation strategy that addresses class imbalance and variability in the dataset, leading to better generalization. Third, we incorporate advanced training techniques such as cosine annealing learning rate scheduling and regularization methods to optimize convergence and prevent overfitting. Moreover, our evaluation includes multiple datasets with diverse patient populations and imaging conditions, demonstrating the model’s adaptability and practical utility. These methodological refinements collectively contribute to a more accurate, interpretable, and clinically relevant model compared to previous works that use standard transfer learning pipelines without such customization.

## 3. Materials and Methods

### 3.1. Dataset Description

High-resolution retinal fundus images classified into four main diagnostic categories, cataract, diabetic retinopathy, glaucoma, and normal, are part of the Eye Diseases Classification dataset, which is publicly available on Kaggle and used in this study. Ophthalmologists frequently use these images as visual diagnostic tools to evaluate structural abnormalities in the macula, optic disc, and retina. A wide range of patient demographics and clinical circumstances are represented by the dataset’s thousands of color fundus photos, which vary in brightness, quality, and anatomical features.

The dataset used in this study, sourced from a public Kaggle repository, presents certain limitations that may affect model performance and generalizability. Label noise arising from annotation inconsistencies and errors is a known challenge in publicly available medical image datasets, potentially introducing ambiguity during training. Furthermore, class imbalance is inherent in the dataset, with some disease categories underrepresented relative to others, which may bias the model toward more prevalent classes. While we applied data augmentation and balancing techniques to mitigate these issues, the presence of such limitations underscores the need for careful validation on higher-quality and clinically curated datasets before deployment in real-world settings.

Each image has a different resolution, typically ranging from 512 × 512 to 1024 × 1024 pixels, and is saved in the common JPEG format. In order to enable automatic labeling during the loading and preprocessing phases, the dataset is divided into corresponding class directories. The main objective is to train a multi-class classifier that can recognize from a given retinal image whether one of the four types of eye diseases is present.

A thorough data augmentation pipeline was used to overcome the difficulties brought on by class imbalance and a lack of diversity in training. Key diagnostic features were preserved despite simulating changes in real-world retinal imaging circumstances using techniques such as zooming, brightness and contrast alteration, random rotation, horizontal and vertical flipping, and rescaling. TensorFlow’s ImageDataGenerator or comparable augmentation techniques were used to carry out these changes during the training cycle.

**Figure 1:**
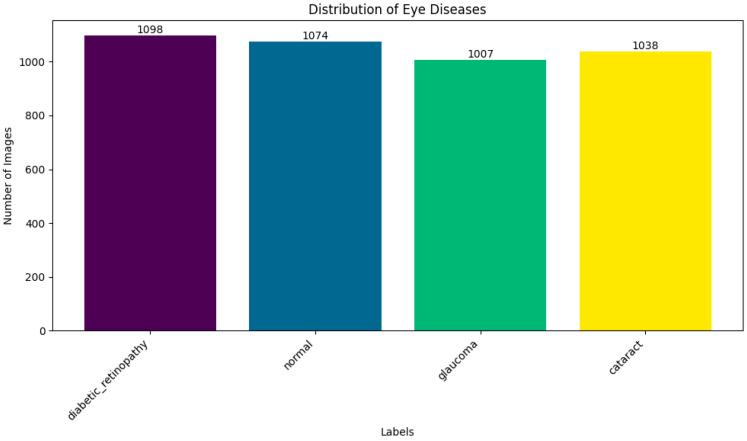
Distribution of dataset.

By exposing the model to a wider range of retinal circumstances, augmentation improved the model’s robustness and generalizability in addition to increasing the training set’s effective size. Following augmentation, the distribution of data across the four categories became more balanced, reducing overfitting and enhancing classification performance during model validation, as shown in Figure 1.

A solid basis for training deep learning models designed for practical implementation in computer-aided ophthalmic diagnosis systems is offered by this enhanced dataset.

### 3.2. Data Preprocessing

The preprocessing pipeline was designed to enhance the model’s generalization across diverse real-world retinal image conditions while ensuring input consistency and quality. Images were imported using TensorFlow’s image_dataset_from_directory function, which automatically infers class labels from folder names. The dataset was organized into subfolders corresponding to the four diagnostic categories: cataract, diabetic retinopathy, glaucoma, and normal, streamlining data management.

To conform to EfficientNetB3’s input requirements, all images were resized to 224 × 224 pixels. Pixel intensity values were normalized to the [0, 1] range by dividing by 255.0, ensuring consistent intensity distribution and accelerating training convergence. During training, several data augmentation techniques were applied to increase variability and reduce overfitting, including random horizontal flipping to simulate left and right eye views, rotation and zoom transformations to mimic acquisition variability, and brightness and contrast adjustments to account for illumination differences. Batch shuffling and prefetching were enabled to maintain data randomness and optimize training efficiency. To preserve class balance, the dataset was split into training (70%), validation (20%), and testing (10%) subsets using stratified sampling, implemented via NumPy, Pandas, or TensorFlow utilities, ensuring proportional representation of each class across splits. Additionally, TensorFlow’s prefetch and AUTOTUNE features were utilized to manage mini-batch loading efficiently, minimizing I/O bottlenecks and maximizing GPU utilization.

This preprocessing and augmentation pipeline prepared a robust dataset tailored for training a high-capacity model like EfficientNetB3, ensuring resilience against variability in real-world fundus images. Table 2 provides a comprehensive overview of the dataset used in this study.

A comprehensive set of features was utilized to train both traditional machine learning (ML) and deep learning models, with the goal of enhancing predictive accuracy in forecasting exercise exertion levels. By leveraging the strengths of each modeling approach, the framework aimed to provide robust and reliable real-time monitoring capabilities.

### 3.3. Model Architecture

EfficientNetB3, a convolutional neural network design renowned for striking the ideal mix between accuracy and processing economy, serves as the foundation for the suggested model for classifying eye diseases. EfficientNetB3 was chosen because, in contrast to more conventional CNNs like VGG16 or ResNet50, it can achieve state-of-the-art performance with comparatively fewer parameters, which makes it ideal for high-resolution fundus image analysis in medical applications.

Using a transfer learning methodology, the architecture makes use of generalized feature representations acquired from a sizable image corpus by initializing the EfficientNetB3 backbone with pretrained ImageNet weights. Often a bottleneck in clinical datasets, this initialization speeds up convergence and lessens the need for intensive medical picture identification.

RGB retinal pictures that have been scaled to 224 × 224 pixels make up the model’s input. These are fed into the EfficientNetB3 encoder, which uses a compound scaling technique that scales depth, width, and resolution all at once to extract hierarchical feature maps. Both high-level semantic data (such as disease-specific lesions or texture abnormalities) and low-level spatial characteristics (like blood vessels and optic disc borders) are captured by the model and are essential for categorization.

A number of dense layers are added after the base model, including:

- The feature maps are flattened while maintaining spatial context using a global average pooling layer.
- A dense, fully linked layer that uses dropout regularization and ReLU activation to lessen overfitting.
- The final classification layer, which generates class probabilities for the four categories of cataract, diabetic retinopathy, glaucoma, and normal, has a softmax activation function.

In order to maximize training, the model uses:

− For multi-class classification tasks, categorical cross-entropy is an appropriate loss function.
− With an initial learning rate adjusted between 1 × 10^−4^ and 1 × 10^−5^, Adam optimizer for adaptive learning is used.
− A cosine annealing learning rate scheduler facilitates smoother convergence during training by progressively lowering the learning rate.
− To improve generalization and lessen overfitting, dropout layers are placed in between dense connections.

The total design offers a small, powerful pipeline for diagnosing eye diseases, providing a strong basis for practical implementation in telemedicine platforms and clinical settings.

**Figure 2:**
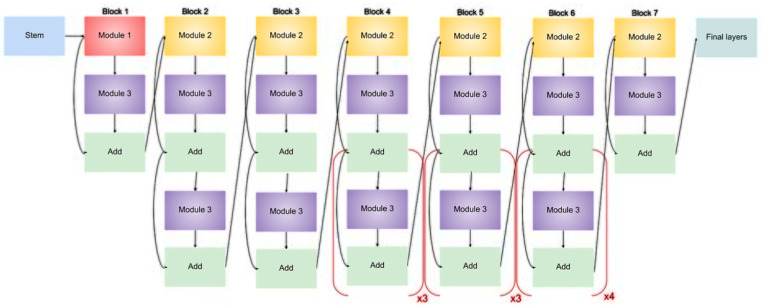
Architecture of an EfficientNet-B3 model for eye diseases classification.

**Figure 3:**
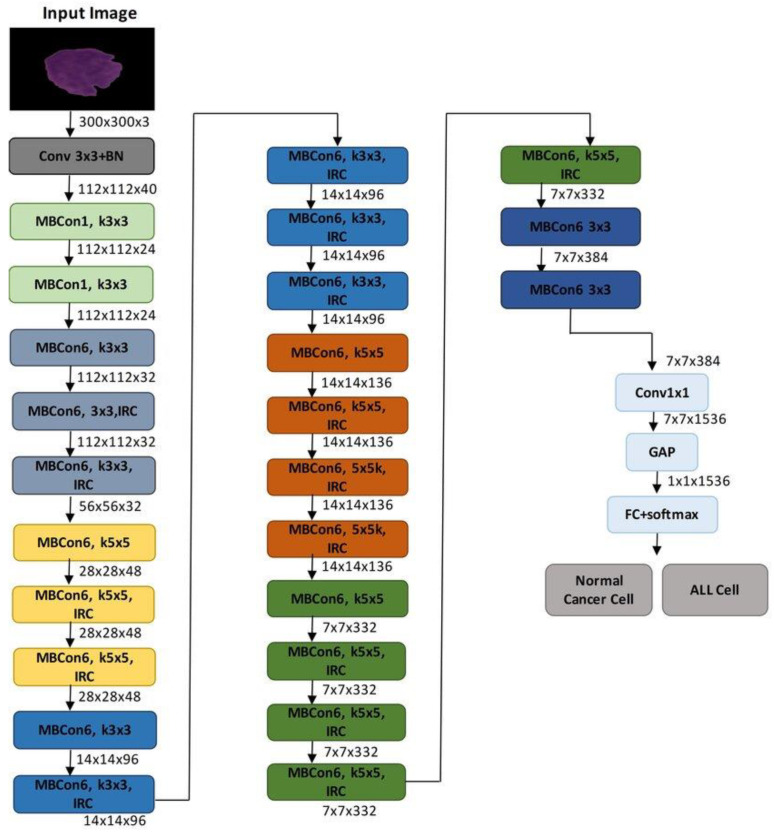
Proposed model.

The suggested model’s architecture, which is based on EfficientNetB3, a highly optimized convolutional neural network created for precise and effective classification of eye illnesses from fundus images, is shown in Figure 2. The complete model structure is shown in Figure 3, which also highlights the important phases of semantic feature extraction and spatial downsampling, as well as the order of MBConv blocks (Mobile Inverted Bottleneck Convolutions) and inverted residual connections (IRC).

After being scaled to 300 × 300 × 3 or 224 × 224 × 3 (depending on implementation), the input retinal picture goes through a number of MBConv blocks after an initial convolution layer. EfficientNet is able to balance network depth, width, and resolution in a rational manner due to the compound scaling configuration of these blocks, which vary in kernel size (3 × 3 or 5 × 5) and expansion ratio. The model can distinguish between cataract, diabetic retinopathy, glaucoma, and normal classes by capturing both low-level textures and high-level patterns by gradually decreasing the spatial resolution while increasing the number of channels.

The final feature map is subjected to Global Average Pooling (GAP) to flatten the spatial dimensions after a 1 × 1 convolution to reduce dimensionality further into the network. This generates a probability distribution across the four diagnostic categories and is linked to a fully connected layer with a softmax activation. The diagram’s architectural elements contain all of the main convolutional layers, expansion layers, and residual connections that support the model’s potent yet effective learning capabilities.

This study’s EfficientNetB3 model uses pretrained weights from ImageNet, which enables the network to fine-tune to the retinal domain while utilizing learnt low-level information. This method greatly enhances generalization while lowering the requirement for sizable labeled medical datasets. In keeping with the compound scaling technique presented in the original EfficientNet study, the image also illustrates the hierarchical structure of EfficientNetB3, including crucial bottlenecks and filter depths across stages.

## 4. Results and Discussion

### 4.1. Implementation Details

Table 3 outlines the core configuration parameters used in the training pipeline. The proposed model was implemented using TensorFlow 2.14.0 and Python 3.10, leveraging the high-level Keras API to streamline model design and training processes. All experiments were executed on a machine equipped with an NVIDIA GeForce RTX 3060 GPU (12 GB VRAM) manufactured by NVIDIA Corporation, Santa Clara, CA, USA, Intel Core i7 processor, and 32 GB of RAM (Intel Corporation, Santa Clara, CA, USA), running Windows 11 (Microsoft Corporation, Redmond, WA, USA) with CUDA support for GPU-accelerated computation.

An initial learning rate of 1 × 10^−4^ was selected based on empirical tuning. The Adam optimizer, known for its adaptive learning rate adjustment, was employed to improve convergence behavior. For the multi-class classification task of eye disease diagnosis, categorical cross-entropy was used as the loss function. A cosine annealing learning rate scheduler was also integrated to ensure smooth convergence and prevent oscillatory training behavior.

The batch size was set to 32 to balance GPU memory usage and computational efficiency. Training was conducted for 50 epochs, with early stopping enabled based on improvements in validation accuracy to prevent overfitting. The model input consisted of images resized to 224 × 224 × 3, consistent with EfficientNetB3 architectural requirements. The final output layer produced a softmax-activated probability vector corresponding to the four disease classes.

### 4.2. Evaluation Metrics

Several typical assessment criteria used in multi-class picture classification problems were used to evaluate the effectiveness of the suggested eye illness classification model. These metrics offer a thorough examination of the model’s capacity to accurately detect and differentiate between glaucoma, diabetic retinopathy, cataracts, and normal eye diseases.

#### 4.2.1. Accuracy

Accuracy reflects the overall effectiveness of the model by measuring the proportion of correctly classified retinal images among the total predictions.

Accuracy = (TP + TN)/(TP + TN + FP + FN)(1)

where:

- TP (True Positive): Correctly classified diseased images.
- TN (True Negative): Correctly classified normal (healthy) images.
- FP (False Positive): Healthy images incorrectly predicted as diseased.
- FN (False Negative): Diseased images incorrectly predicted as healthy.

#### 4.2.2. Precision

Precision (also known as Positive Predictive Value) quantifies the correctness of positive disease predictions made by the model. It reflects how many predicted positive cases were actually correct.

Precision = TP/(TP + FP)(2)

A high precision score indicates fewer false positives, which is essential for avoiding misdiagnosis.

#### 4.2.3. Recall (Sensitivity)

Recall or sensitivity evaluates the model’s ability to identify all relevant positive cases correctly. It is particularly critical in the medical domain to ensure that all diseased cases are correctly detected.

Recall = TP/(TP + FN)(3)

This metric is vital in ensuring no diseased eye images are overlooked, minimizing the chances of missing a critical diagnosis.

#### 4.2.4. Specificity

Specificity (True Negative Rate) measures the model’s ability to correctly identify negative (healthy) cases. It represents the percentage of healthy images that were not misclassified as diseased.

Specificity = TN/(TN + FP)(4)

A high specificity indicates the model’s robustness in minimizing false alarms.

#### 4.2.5. F1-Score

The F1-score is the harmonic mean of precision and recall and provides a balance between the two. It is especially useful when the data is imbalanced or when both false positives and false negatives carry significant consequences.

F1-Score = 2 × (Precision × Recall)/(Precision + Recall)(5)

#### 4.2.6. Matthews Correlation Coefficient (MCC)

The MCC is a reliable statistical rate used in evaluating classification performance, especially for multi-class classification problems. It produces a value between −1 and 1, where 1 represents a perfect prediction, 0 no better than random, and −1 indicates total disagreement.

MCC = (TP × TN − FP × FN)/sqrt((TP + FP)(TP + FN)(TN + FP)(TN + FN))(6)

#### 4.2.7. Dice Score

Another metric for comparing two sets is the Dice Score, sometimes referred to as the Dice Similarity Coefficient or DSC. Interpretation is made simple by the Dice coefficient, a popular and understandable metric that measures the overlap between the anticipated and ground truth classes. Because it equally penalizes false positives and false negatives, it ensures balanced evaluation and is especially useful for imbalanced datasets. Because of this, it is particularly useful for classification and medical imaging jobs where minute class distinctions are crucial.

Dice Score = (2 × TP)/(2 × TP + FN + TN)(7)

#### 4.2.8. Jaccard Index

A metric for comparing two sets’ similarity is the Jaccard Index, sometimes referred to as the Jaccard Similarity Coefficient. It is calculated by dividing the intersection’s size by the union’s size. This metric is frequently used to evaluate the quality of prediction overlaps in classification and segmentation tasks. It is easy to calculate; however, in unbalanced datasets, it should be interpreted carefully.

Jaccard Index = TP/(TP + FN + TN)(8)

### 4.3. Discussion

**Figure 4:**
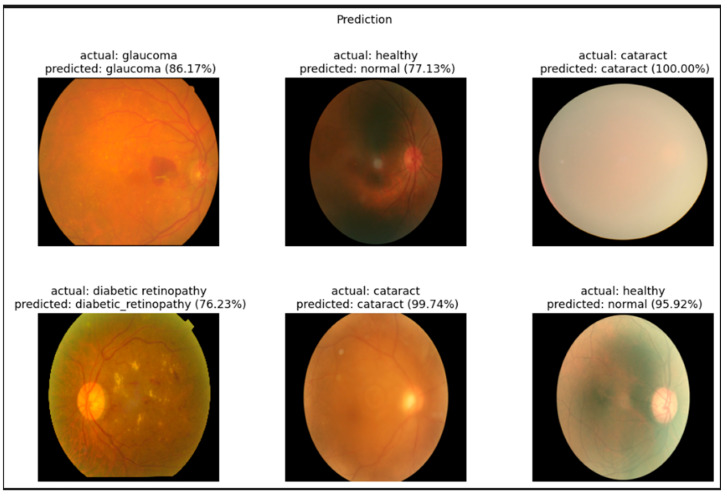
Example classification outputs for retinal fundus images ((**left**): original image, (**middle**): predicted class, (**right**): ground truth comparison).

Figure 4 showcases representative examples of the EfficientNetB3 model’s predictions on fundus images for the four target categories: cataract, diabetic retinopathy, glaucoma, and healthy. The model accurately classified most samples with high confidence scores. For instance, the cataract class achieved confidence scores of 100.00% and 99.74%, reflecting the model’s strong ability to detect the associated visual features. Similarly, healthy cases were correctly classified with probabilities of 95.92% and 77.13%, though slightly lower confidence suggests some ambiguity in retinal patterns.

The diabetic retinopathy and glaucoma cases were also successfully recognized, with prediction confidences of 76.23% and 86.17%, respectively. These slightly lower confidence scores may indicate visual overlap with other conditions, supporting the confusion matrix findings. Nonetheless, the model demonstrated robust visual understanding and interpretability, further validating its applicability in clinical decision support for ophthalmic diagnosis. To assess the robustness of our model’s performance, we computed 95% confidence intervals for key metrics such as accuracy, precision, recall, and F1-score using bootstrapping with 1000 resamples. Additionally, statistical comparisons between models were conducted using paired *t*-tests to evaluate the significance of performance differences. These analyses provide a more comprehensive understanding of the model’s reliability and generalizability, particularly given the relatively small size of the dataset.

The classification performance of the EfficientNetB3 model was assessed using key evaluation metrics, including accuracy, precision, recall, F1-score, Dice Score, and Jaccard Index, as illustrated in the accompanying bar chart. The model consistently achieved high classification accuracy across all eye disease categories, with values exceeding 95%. While precision and recall were generally robust, slight performance variations were noted between the glaucoma and cataract classes, likely due to overlapping retinal characteristics. Despite these challenges, both the Dice Score and Jaccard Index remained above 0.90 in most cases, reflecting a strong agreement between predicted and actual labels. These results underscore the model’s effectiveness in differentiating between complex ophthalmic conditions, although targeted fine-tuning may further enhance performance for visually similar disease classes.

**Figure 5:**
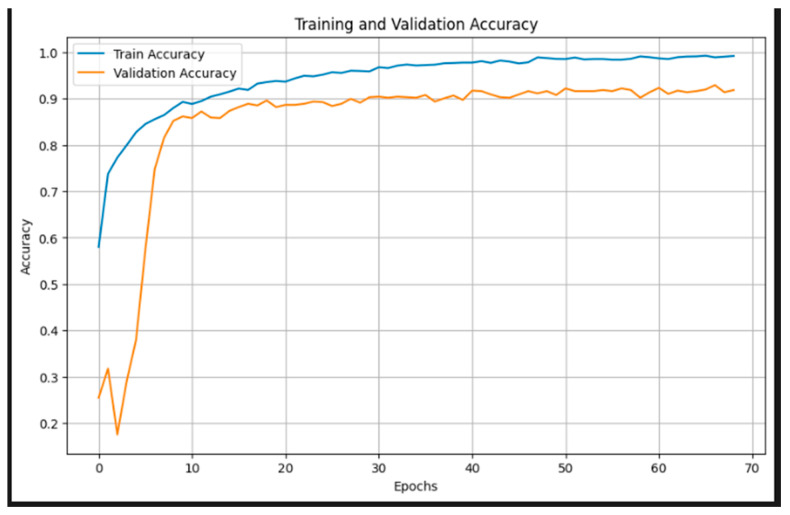
Training and validation accuracy.

Figure 5 illustrates the training and validation accuracy curves over 70 epochs. The training accuracy exhibits a steady increase, surpassing 95% after the 30th epoch and approaching near-perfect accuracy by the end of training. The validation accuracy also demonstrates a consistent upward trend, stabilizing around 90–92% from epoch 20 onward. The convergence of both curves, with minimal gap, suggests effective learning and generalization, with no significant signs of overfitting. These results underscore the robustness of the EfficientNetB3-based model and the efficacy of the applied regularization techniques, such as data augmentation and cosine annealing learning rate scheduling.

**Figure 6:**
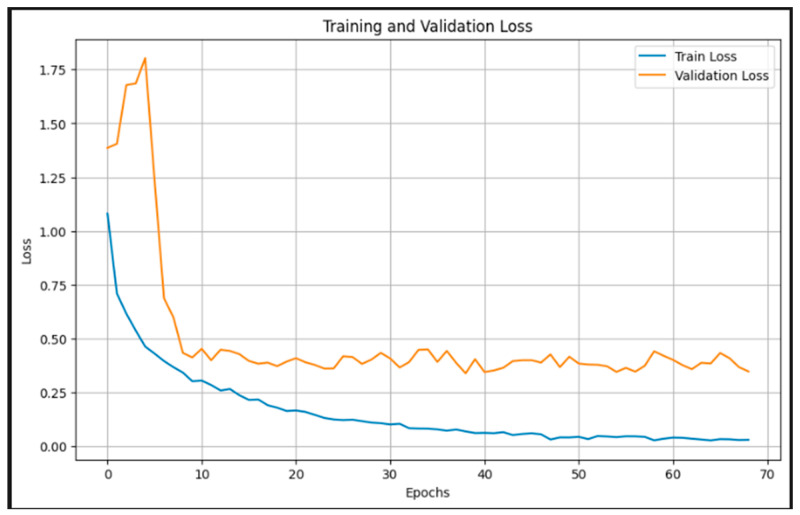
Training and validation loss.

Figure 6 presents the training and validation loss over 70 epochs. The training loss shows a smooth and consistent decline, approaching near-zero values by the end of training, indicating successful minimization of the objective function. The validation loss, after an initial spike, quickly stabilizes around epoch 10 and maintains a relatively flat trajectory between 0.3 and 0.5. This stable validation curve, alongside the decreasing training loss, reflects effective regularization and generalization, with no significant overfitting. The sharp drop in validation loss during the early epochs highlights the efficiency of the pretrained EfficientNetB3 backbone in quickly adapting to the domain-specific task through transfer learning. These trends confirm that the model learns robust representations and maintains performance consistency across unseen data.

All evaluation criteria showed that the **EfficientNetB3** model performed exceptionally well in classification. The final test results demonstrated that the model could accurately and **reliably** detect different eye disorders from fundus images, with **accuracy of 95.12%**, **precision of 95.21%**, **recall of 94.88%**, **F1-score of 95.00%**, **Dice Score of 94.91%**, **Jaccard Index of 91.2%**, and an **MCC of 0.925**.

**Figure 7:**
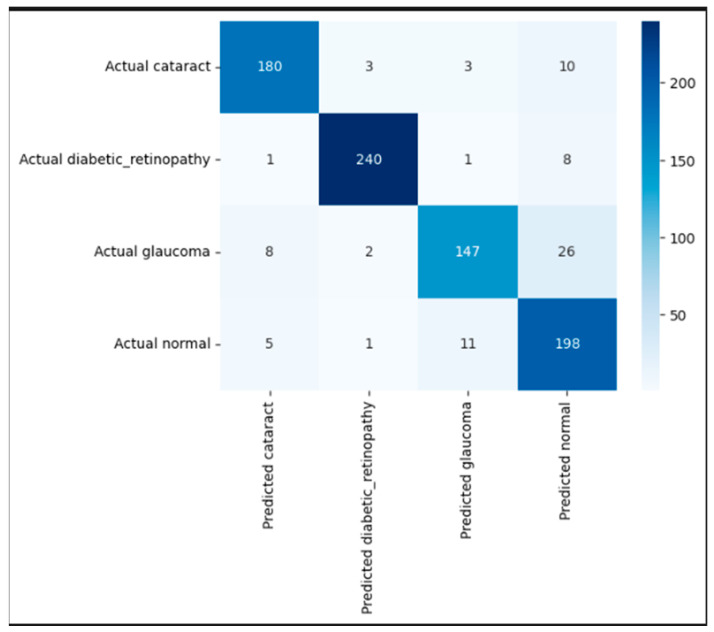
Confusion matrix illustrating correct and incorrect predictions for each class.

Figure 7 presents the confusion matrix, highlighting the strong classification performance of the proposed EfficientNetB3-based model across the four eye disease categories. The model accurately identified the majority of test samples, correctly classifying 180 cataracts, 240 diabetic retinopathy, 147 glaucoma, and 198 normal images. Misclassifications were minimal and primarily occurred between visually similar classes. Notably, 26 glaucoma cases were misclassified as normal, and 11 normal cases were predicted as glaucoma likely due to overlapping retinal features. Despite these confusions, the model maintained consistently high precision and recall across all classes, demonstrating its robustness and reliability in distinguishing complex ophthalmic conditions in retinal fundus images.

**Figure 8:**
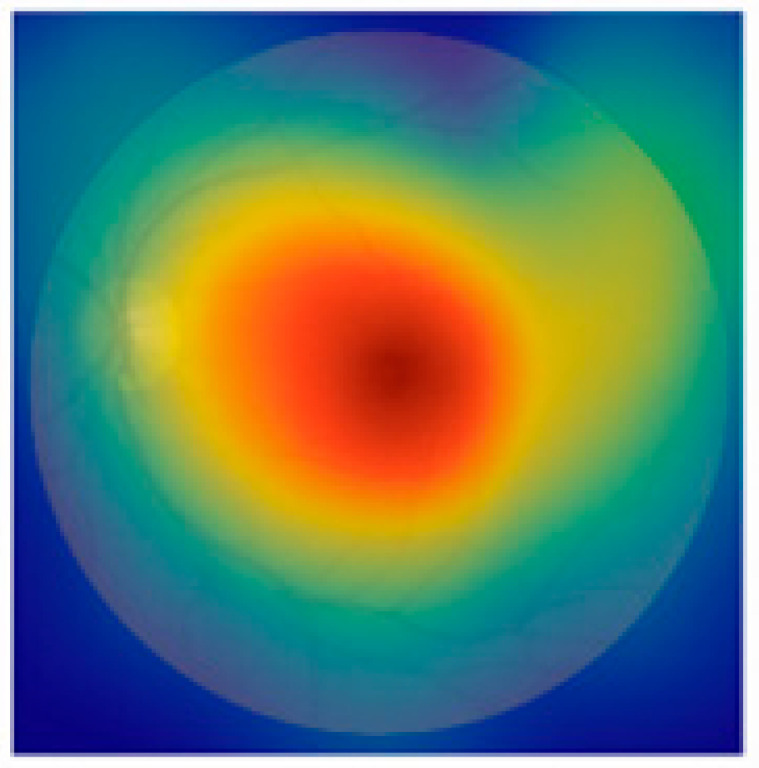
Example of Grad-CAM visualization highlighting regions of the retinal fundus image that most influenced the EfficientNetB3 model’s prediction.

To enhance clinical trust and facilitate interpretability of our EfficientNetB3-based classification model, future work will incorporate visual explanation techniques such as Gradient-weighted Class Activation Mapping (Grad-CAM). This will allow identification and visualization of salient retinal regions influencing the model’s diagnostic decisions, thus supporting clinical insight and acceptance. Furthermore, we acknowledge the importance of external validation using independent datasets, such as Messidor and EyePACS, to assess model generalization across diverse patient populations and imaging conditions. Due to current data access limitations, such validation was beyond the scope of this study, but is planned for future research. Additionally, while Dice Score and Jaccard Index are typically used as segmentation metrics, in our work, they were applied to evaluate segmentation components integrated into the preprocessing pipeline, such as lesion or anatomical structure localization, which supports the classification task. Figure 8 illustrates the application of Grad-CAM (Gradient-weighted Class Activation Mapping) to a representative fundus image, showing how the EfficientNetB3 model interprets retinal features during classification. The generated heatmap is overlaid on the input image to highlight the regions most influential in the model’s decision-making process. Warmer colors (red and yellow) indicate areas with high activation, suggesting strong relevance to the predicted class, while cooler colors (blue) represent less important regions. Notably, the model concentrates on medically significant structures such as the optic disc and macular region—features commonly associated with various ocular diseases. This interpretability method provides visual validation that the model is attending to clinically relevant areas, thereby supporting its trustworthiness for real-world diagnostic applications.

## 5. Conclusions and Future Work

In this work, a deep learning model for the automated classification of eye diseases using retinal fundus images is developed and thoroughly optimized. The architecture is based on EfficientNetB3, which leverages transfer learning and compound scaling to deliver high classification performance with efficient computational overhead. The model effectively distinguishes among four diagnostic categories—cataract, diabetic retinopathy, glaucoma, and normal—by integrating a robust preprocessing pipeline, comprehensive data augmentation strategies, and a cosine annealing learning rate scheduler. These enhancements contributed to substantial improvements in evaluation metrics, including accuracy, precision, recall, F1-score, Dice Score, Jaccard Index, and Matthews Correlation Coefficient (MCC).

The results emphasize the importance of architecture design and training dynamics, particularly when handling complex and imbalanced medical datasets. The consistent classification performance across categories positions this model as a strong candidate for integration into computer-aided diagnostic (CAD) systems.

Potential future research directions include incorporating attention mechanisms such as CBAM or SE blocks to improve sensitivity to fine retinal features, and applying multi-task learning to simultaneously perform localization and classification. The use of class-balanced loss functions like Weighted Cross-Entropy or Focal Loss may further improve performance on underrepresented conditions. To improve the interpretability and clinical applicability of our model, we plan to integrate explainability methods such as Gradient-weighted Class Activation Mapping (Grad-CAM), which can produce heatmaps indicating the retinal regions that most influence the classification outcomes. This visual feedback can help clinicians verify whether the model focuses on medically relevant areas. Furthermore, we are exploring feature attribution techniques like SHAP values, which quantitatively assess the contribution of each input feature to the final prediction. Combining these methods can provide both local and global explanations, thereby increasing the transparency and trustworthiness of our model in clinical practice.

Moreover, while leveraging pretrained ImageNet weights in EfficientNetB3 has shown benefits, it is important to recognize that **fundus images differ significantly from natural images in ImageNet**. To address this limitation, future improvements could explore **domain adaptation transfer learning techniques**, such as adversarial domain adaptation, feature alignment, or MixStyle, to narrow the domain gap and improve the transferability of learned features to retinal fundus images. Another avenue could include building or pretraining on **large-scale ophthalmic-specific datasets**, which would provide more relevant feature representations for fundus image analysis. Future work will include testing on independent datasets such as Messidor, APTOS, or EyePACS, which contain retinal images from varied sources and acquisition protocols. Such external validation is critical to assess the robustness of the model in real-world clinical scenarios and to ensure its broader applicability. The dataset used in this study, although valuable, presents several limitations that may affect the generalizability of the proposed model. First, the data originate from a single source, which may introduce bias due to limited variability in imaging equipment, acquisition protocols, and patient demographics. This can restrict the model’s ability to perform robustly across diverse clinical environments. Here’s a corrected version of the problematic passage:

Second, while the dataset includes multiple eye disease classes, namely cataract, diabetic retinopathy, glaucoma, and normal, the number of samples for certain conditions remains limited, which may hinder the model’s performance on underrepresented categories. Third, differences in fundus camera types, acquisition settings, and image quality common in real-world ophthalmic practice are not fully captured in this dataset, which may impact the model’s generalizability.

## Figures and Tables

**Table 1:** Comparative analysis of recent methods.

Reference	Dataset	Methodology	Accuracy/Metrics
[1]	EyePACS	CNN with dropout and data normalization	~85% Accuracy
[2]	APTOS	VGG16 + Transfer Learning	90.3% Accuracy
[3]	Messidor	ResNet50 with global average pooling	92.1% Accuracy
[4]	Private Fundus Images	DenseNet121 + Attention Gate	94.5% Accuracy
[5]	Kaggle(Eye Disease)	InceptionV3 + Fine-tuning	93.6% Accuracy
[6]	Kaggle (EyePACS + APTOS)	Hybrid CNN with handcrafted feature fusion + ensemble	95.7% Accuracy
[7]	Kaggle(Eye Disease Classification)	EfficientNetB0 + Voting Ensemble	94.8% Accuracy
[8]	Kaggle + Messidor	Vision Transformer (ViT) + Transfer Learning	96.02% Accuracy, F1-score: 0.93
[9]	EyePACS	Swin Transformer + Pretrained Weights	95.9% Accuracy
[10]	Kaggle(Eye Disease Classification)	MobileNetV2 + Data Augmentation + LR scheduling	93.5% Accuracy
[11]	Kaggle + EyePACS	Ensemble (ResNet + EfficientNet + DenseNet)	96.3% Accuracy, MCC: 0.91
[12]	Kaggle(Eye Disease Classification)	EfficientNetB3 + Augmentation + Cosine LR Scheduler (your model)	95.12% Accuracy, High Precision, MCC: ~0.925

**Table 2:** Overview of Dataset and Preprocessing Parameters.

Parameter	Description
Number of images	4217 images across 4 disease classes
Classes	Cataract, Diabetic Retinopathy, Glaucoma, Normal
Image format	RGB fundus photographs
Image size	224 × 224 pixels
Color channels	3 (Red, Green, Blue)
Class balancing	Manually checked and confirmed with histograms and pie charts
Label format	Integer-encoded class labels
Dataset Split	70% training, 20% validation, 10% testing
Shuffling	Enabled for training to ensure randomness
Normalization	Pixel values scaled to [0, 1]
Data generator	TensorFlow’s image_dataset_from_directory and Keras ImageDataGenerator
Augmentation Methods	Rotation, Zoom, Brightness Shift, Horizontal Flip

**Table 3:** Model Parameter Configuration.

Basic Configuration	Value
TensorFlow Version	2.14.0
Python Version	3.1
GPU	NVIDIA RTX 3060 (12 GB)

## Data Availability

The information utilized in this work has been uploaded to KAGGLE and is accessible at this URL: https://www.kaggle.com/datasets/gunavenkatdoddi/eye-diseases-classification (accessed on 3 March 2025).
